# Rottlerin Stimulates Exosome/Microvesicle Release Via the Increase of Ceramide Levels Mediated by Ampk in an In Vitro Model of Intracellular Lipid Accumulation

**DOI:** 10.3390/biomedicines10061316

**Published:** 2022-06-03

**Authors:** Yessenia L. Molina, David García-Seisdedos, Bohdan Babiy, Milagros Lerma, Javier Martínez-Botas, María J. Casarejos, María T. Vallejo, Diego Gómez-Coronado, Miguel A. Lasunción, Óscar Pastor, Rebeca Busto

**Affiliations:** 1 Servicio de Bioquímica-Investigación, Hospital Universitario Ramón y Cajal, IRYCIS, 28034 Madrid, Spain; yesse1497@gmail.com (Y.L.M.); dgseis@hotmail.es (D.G.-S.); mila93.lerma@gmail.com (M.L.); javier.botas@hrc.es (J.M.-B.); diego.gomez@hrc.es (D.G.-C.); malasuncion@hotmail.com (M.A.L.); 2 Servicio de Bioquímica-Clínica, Hospital Universitario Ramón y Cajal, IRYCIS, 28034 Madrid, Spain; bohdan@hotmail.es; 3 CIBER Fisiopatología de la Obesidad y Nutrición (CIBEROBN), Instituto de Salud Carlos III (ISCIII), 28029 Madrid, Spain; 4 Servicio de Neurobiología, Hospital Universitario Ramón y Cajal, IRYCIS, 28034 Madrid, Spain; m.jose.casarejos@hrc.es; 5 Molecular Imaging and Immunohistochemistry Unit, Hospital Universitario la Paz, IdiPAZ, 28046 Madrid, Spain; teresa.vallejo@salud.madrid.org

**Keywords:** rottlerin, exosome/microvesicle, intracellular lipid trafficking, ceramide, sphingolipid, lysosomal storage disease, AMPK

## Abstract

Exosomes/microvesicles originate from multivesicular bodies that allow the secretion of endolysosome components out of the cell. In the present work, we investigated the effects of rottlerin, a polyphenol, on exosome/microvesicle secretion in a model of intracellular lipid trafficking impairment, and elucidated the mechanism of action. In a model of lipid trafficking impairment in C6 glia cells, rottlerin increased ceramide levels, while decreasing hexosylceramide content. This was accompanied by increased exosome/microvesicle secretion, thereby reducing the concentration of lipids in the endolysosomal compartment. The reduction of hexosylceramide levels by rottlerin was attributed to the increase of β-glucosidase (glucosylceramidase) activity, and the effects of rottlerin were abrogated by β-glucosidase inhibitors such as isofagomine D-tartrate and AMP-deoxynojirimycin. Moreover, treatment with ML-266, a potent activator of the β-glucosidase enzyme, recapitulated the effects of rottlerin on the sphingolipid profile and exosome/microvesicle secretion. Finally, inhibition of AMPK (AMP-activated protein kinase) using compound C prevented both exosome/microvesicle secretion and the elimination of endolysosome lipids, which were promoted by rottlerin. The results showed that the decrease in intracellular lipid deposition induced by rottlerin was mediated by β-glucosidase activation and exosome/microvesicle release via the AMPK pathway. Rottlerin consumption could represent an additional health benefit in lysosomal deposition diseases.

## 1. Introduction

Exosomes/microvesicles are small (40–150 nm in diameter) endosome-derived intraluminal vesicles that originate from large multivesicular endosomes designated multivesicular bodies (MVBs) and are released from cells after MVBs fuse with the plasma membrane [1]. Exosomes/microvesicles play an active role in intercellular communication by transporting a wide range of bioactive molecules between different cells and tissues [2].

Cargo sorting in exosomes/microvesicles biogenesis has been shown to involve the endosomal sorting complex required for transport (ESCRT)-dependent or ESCRT-independent mechanisms [3]. Nonetheless, the pathways involved in these mechanisms may be interconnected [4]. Several lipids and lipid-metabolizing enzymes have been shown to play roles in the formation and release of exosomes/microvesicles [5,6]. In particular, ceramide appears to be essential for exosome/microvesicle secretion [7,8,9,10,11]. Ceramide is a coned-shaped lipid that promotes the coalescence of membrane domains and induces the spontaneous curvature of membranes, promoting domain-induced budding [12]. A decrease in ceramide levels reduces the secretion of exosomes/microvesicles from Oli-neu cells [13]. It has been proposed that ceramide favors the formation of microdomains in areas with high sphingolipid concentrations, followed by coalescence into larger ceramide-rich domains, which induce membrane-budding [13].

We previously reported that curcumin, a hydrophobic polyphenol found in the rhizomes of *Curcuma longa*, stimulated exosome/microvesicle release via an increase in ceramide, allowing the export of lipids out of the cell, and therefore ameliorating the endo/lysosomal lipid accumulation found in lysosomal storage disorders [10]. Rottlerin is the principal polyphenol component of the Kamala plant, and is the red powder that covers the fruits of *Mallotus philippinensis* (Euphorbiaceae), an evergreen tree that grows in the tropical regions of Southeast Asia [14]. Rottlerin has been used for decades as a selective protein kinase C-δ inhibitor [15], although some controversy on this aspect still exists [16]. Moreover, extended treatment of rottlerin increases cellular ceramide concentrations in erythrocytes [17]. However, the potential effects of rottlerin on exosomes/microvesicle release have not been examined.

AMP-activated protein kinase (AMPK) is a ubiquitous energy-sensing enzyme within cells that is critical in maintaining metabolic homeostasis, and it is a central player in glucose and lipid metabolism [18]. Recent studies have reported a connection between the AMPK pathway and exosome/microvesicle secretion [19,20]. Moreover, rottlerin activates the AMPK pathway in different cell types [21,22]. 

In this study, we therefore characterized the role of rottlerin in inducing the release of exosomes/microvesicles in a model of intracellular lipid trafficking impairment, and then identified its possible mechanism of action.

## 2. Materials and Methods

Except as otherwise indicated, all chemicals were purchased from Sigma-Aldrich (Sigma-Aldrich Química, S.A., Madrid, Spain). All compounds were dissolved in dimethyl sulfoxide (DMSO), which was added to the cell culture medium at a final concentration of 0.044%.

### 2.1. Cell Culture

Rat C6 glial cells (American Type Tissue Collection, CCL-107™; Manassas, VA, USA), obtained from C. Paíno (Departamento de Investigación, Hospital Universitario Ramón y Cajal, Madrid, Spain), were cultured in Ham’s F12 medium supplemented with L-glutamine (Fisher Scientific, Madrid, Spain), MEM nonessential amino acids, 10% fetal bovine serum (FBS), and antibiotics (Fisher Scientific, Madrid, Spain) at 37 °C in a 5% CO_2_ atmosphere. Lipoprotein-deficient serum (LPDS) was prepared from FBS by ultracentrifugation at 105,000× *g* for 48 h at a density of 1.21 kg/L.

Human low-density lipoprotein (LDL) was isolated as described previously [23] and labeled with the fluorescent probe, 1,1′-dioctadecyl-3,3,3,3′-tetramethylindocarbocyanineperchlorate (DiI; Fisher Scientific, Madrid, Spain), as previously described [24]. Cell viability was determined using XTT assays (Cell Proliferation Kit; Roche, Mannheim, Germany).

### 2.2. Immunofluorescence Microscopy

Cells were incubated in medium supplemented with 10% LPDS and DiI-LDL (20 µg cholesterol/mL) and incubated without drugs or with 2.5 µM U18666A (Tocris, Bristol, UK) for 16 h on glass coverslips. The cells were then supplemented with or without rottlerin (0.5 µM, 2 µM, 5 µM, or 10 µM) (Acros Organics^®^; Fisher Scientific, Madrid, Spain) or ML266 (50 µM) (Aobious, Gloucester, MA, USA) as indicated, then incubated for the last 4 h. In other experiments, the cells were treated with or without 5 µM rottlerin in combination with or without compound C (10 or 20 µM) and incubated for the last 4 h. The cells were then fixed with 4% paraformaldehyde/PBS for 5 min, mounted for microscopy, and examined using a confocal TCS SP5 microscope (Leica, Wetzlar, Germany). DiI-LDL-cell content images were quantified using NIS-elements Basic Research software (Nikon, Tokyo, Japan).

To study β-galactosidase and β-glucosidase activities, the cells were maintained in LPDS medium for 2 h, then treated with or without rottlerin incubated with 33 µM C12FDG (5-dodecanoylaminofluorescein di-β-D-galactopyranoside, Fisher Scientific, Madrid, Spain) or 33 µM FDGlu (di-β-D-glucopyranoside; Fisher Scientific, Madrid, Spain) for 30 min on glass coverslips. The cells were then fixed with 4% paraformaldehyde/PBS for 5 min, mounted for microscopy, and examined using a Nikon D Eclipse C1 confocal microscope.

### 2.3. Western Blotting

Cells were homogenized in a solution of 50 mM Tris-HCl pH 7.5, 125 mM NaCl, 1% Nonidet P40, 5 mM NaF, 1.4 mM Na_4_O_7_P_2_, 1 mM Na_3_VO_4_, and protease inhibitor (Fisher Scientific, Madrid, Spain). The homogenate was then centrifuged at 600× *g* for 5 min at 4 °C. Protein determination was performed using the bicinchoninic acid protein assay (Pierce Biotechnology, Waltham, MA, USA). Whole cell lysates (20–30 μg) were subjected to 10% SDS-PAGE and transferred to nitrocellulose membranes (Bio-Rad Laboratories, Barcelona, Spain). After blocking, the blots were incubated with the following antibodies: anti-phospho-AMPK (T172) (Cell Signaling Technology, Danvers, MA, USA), anti-AMPK (Cell Signaling Technology), and anti-β-actin (Santa Cruz Biotechnology, Santa Cruz, CA, USA), followed by a secondary antibody conjugated to IRDye 800 CW or IRDye 680 LT (LI-COR, Lincoln, NE, USA). The membranes were then analyzed using the Odyssey Infrared Imaging System (LI-COR). Densitometric analysis was performed using the Odyssey Infrared Imaging System, version 3.0 (LI-COR).

### 2.4. Exosome/microvesicle Isolation and Analysis

Cells were incubated in medium supplemented with 10% LPDS and LDL (60 µg/mL of cholesterol) and exposed to 2.5 µM U18666A for 16 h with or without rottlerin (5 µM and 10 µM) or 50 µM ML266, or supplemented with or without 5 µM rottlerin in combination with or without 2 µM isofagomine D-tartrate (IFG) (Cayman Chemical, Ann Arbor, MI, USA) or 2 µM AMP-deoxynojirimycin (AMP-DNM) (Cayman Chemical) for the last 4 h. In other experiments, the cells were treated with or without 5 µM rottlerin in combination with or without compound C (10 µM or 20 µM) for the last 4 h. Exosomes/microvesicles were prepared as previously described [10]. Briefly, after cell treatment, the medium was collected on ice, centrifuged at 800× *g* for 10 min to sediment cells, and then at 12,000× *g* for 30 min to sediment debris. Exosomes/microvesicles were harvested from the remaining supernatant by ultracentrifugation at 100,000× *g* for 2 h. The pellet containing exosomes/microvesicles was resuspended in PBS.

For each condition, equal numbers of cells were seeded in 75 cm^2^ culture flasks, and at the end of the experiment, exosomes/microvesicles were isolated from the culture medium. The exosome/microvesicle concentration was determined by nanoparticle tracking analysis using NanoSight LM10, version 3.2).

Western blot analysis was performed as previously described [25]. Exosome/microvesicle samples were loaded with as much volume in gel wells as possible. Samples were resolved using 10% SDS-PAGE and transferred to nitrocellulose membranes (Bio-Rad Laboratories). The blots were blocked, probed with specific antibodies (anti-flotillin-1 (BD Biosciences, Franklin Lakes, NJ, USA) and anti-CD63 (Bioss Antibodies, Woburn, MA, USA)), and incubated with secondary antibodies conjugated to IRDye 800CW or IRDye 680LT (LI-COR) in the dark. The membranes were then analyzed and quantified using the Odyssey Infrared Imaging System (LI-COR).

### 2.5. Lipidomic Analysis

Cells were cultured in medium containing 10% LPDS for 2 h and incubated for 4 h without (control) or with rottlerin (2 µM, 5 µM, and 10 μM), or with or without 5 μM rottlerin supplemented with 2 µM IFG or 2 µM AMP-DNM; without (control) or with 50 μM ML266. The lipidomic analysis was conducted following previously described methodology [26]. The lipid fraction from the cells (500 µg of protein) was extracted according to the method of Folch et al. [27]. One internal standard was added for each lipid class: Cer 37:1 (d18:1/19:0), HexCer 33:1 (d18:1/15:0), and SM 30:1 (d18:1/12:0) (Avanti Polar Lipids, Alabaster, AL, USA) before lipid extraction of all samples to obtain the relative molar quantifications. The lipid extracts were dried and suspended in 250 µL of acetonitrile/isopropanol (1:1), then injected into an LC Eksigent UltraLC-100 (AB-Sciex, Vaughan, ON, Canada). The lipid species were separated using a Kinetex C18 column (100 × 2.1 mm, 1.7 µm; Phenomenex, Macclesfield, UK) maintained at 55 °C. Elution was carried out using a system consisting of solvent A (60% acetonitrile in water, 10 mM ammonium formate) and solvent B (90% isopropyl alcohol in acetonitrile and 10 mM ammonium formate) and a linear gradient from 60% A to 100% B for 12 min and 100% B to 60% A for 8 min at a flow rate of 0.4 mL/min. The detection of lipid species was conducted following a targeted approach setting MRM transitions for each lipid species at their retention times. The detection of lipid classes was performed using a QTrap 4000 (AB-Sciex) with Analyst 1.6.2 software (AB-Sciex). Nitrogen was used as the drying gas at a temperature of 500 °C, the curtain gas was set at 30 psi, and the ion source gas was set at 50 psi. The injection volume was 5 µL. The LC-MS/MS peak chromatograms were processed using Skyline software version 4.1 [28,29]. The lipid species were quantified according to a matrix-matched single-point calibration with internal standardization schema as previously described [26]. The level of absolute quantification achieved by our methodology was level 3 following the Lipidomics Standard Initiative guidelines (Initiative LS. https://lipidomics-standards-initiative.org/guidelines/lipid-species-quantification, accessed on 19 April 2022).

### 2.6. Determination of β-Galactosidase and β-Glucosidase Activities by Flow Cytometry

To measure β-galactosidase and β-glucosidase activities by flow cytometry, we used the C12FDG fluorogenic substrate as previously described [30] and FDGlu, respectively. For the assays, cells were maintained in LPDS medium for 2 h, with or without rottlerin and C12FDG or FDGlu (33 µM) for the last 30 min. In other experiments, cells were cultured in LPDS medium in the presence of AMP-DNM or IFG (2 µM each) for 2 h, then were treated with or without rottlerin, with the substrate for β-galactosidase or β-glucosidase enzymes added for the last 30 min. At the end of incubation, the cells were analyzed using a FACScan-Flow cytometry system (BD Biosciences, San Jose, CA, USA). Light-scatter parameters were used to exclude dead cells and subcellular debris from the analysis. The C12-fluorescein or di-β-D-glucopyranoside fluorescent signal was measured on the FL1 detector, and both enzymatic activities were estimated using the median intensity of the fluorescence (MIF) of the cell population.

### 2.7. RNA Isolation and Quantitative RT-PCR (qRT-PCR)

Total RNA from C6 cells was extracted using TRI-Reagent (MRC Molecular Research Center, Cincinnati, OH, USA) according to the manufacturer’s recommendations, and reverse-transcribed with random hexamers and oligo dT primer using the PrimeScript RT reagent kit (Takara Bio, Kusatsu, Japan). The qRT-PCR amplification was performed using a LightCycler 480 system using the SYBR Green I Master kit (Roche Applied Science, Penzberg, Germany). The amplification protocol consisted of an initial denaturation step at 95 °C for 5 min, followed by 45 cycles at 95 °C for 10 s, 60 °C for 10 s, and 72 °C for 10 s. Melting curves were evaluated for each gene, and the amplification products were separated on 2% agarose gels to confirm the presence of a single band. The efficiency of the reaction was evaluated by amplifying serial dilutions of cDNA (1:10, 1:100, 1:1000, and 1:10,000). We ensured that the relationship between the threshold cycle (Ct) and the log(RNA) was linear (−3.6 < slope < 3.2). All analyses were performed in triplicate. The number of copies of the target gene was normalized against the media of housekeeping genes glyceraldehyde-3-phosphate dehydrogenase (Gapdh) and ribosomal protein lateral stalk subunit P0 (Rplp0). The primers used in qRT-PCR are shown in Table S1 in Supplementary Materials.

### 2.8. Statistical Analysis

Data are shown as mean ± SEM. The results of immunocytochemistry, flow cytometry, and sphingolipid composition (group and species) were analyzed by two-way (treatment and day) analysis of variance (ANOVA) followed by the Bonferroni post test. In other analyses, the effects of treatment time on sphingolipid composition or XTT results were assessed by one-way repeated-measures ANOVA followed by the Bonferroni post hoc test or t-test for the number of total particles. Means among groups were considered significantly different when the *p* value was less than 0.05.

## 3. Results

### 3.1. Rottlerin Reduces Lysosomal LDL-Cholesterol Accumulation

The rat glioblastoma and astrocytoma cell line, C6, was used in this study. In the central nervous system, astrocytes are one of the sources of lipid synthesis and secretion [31]. These cells have the ability to intensely synthesize sphingolipids, and therefore represent a suitable cell model to search for ways to ameliorate perturbations caused by intracellular lipid trafficking disruption relevant in the central nervous system. The effects of increasing doses of rottlerin in C6 cells treated with U18666A, an inhibitor of intracellular cholesterol trafficking, were analyzed [32].

Although short incubation periods were used throughout the study, we first liked to determine if the different compounds under study affected the viability of C6 cells. Treatment of cells with or without 2.5 μM U18666A (control), rottlerin (2 μM, 5 μM, and 10 μM), or ML266 50 μM (activators of β-glucosylceramidase or β-glucosidase) for 4 h had no effect on cell viability as measured by the XTT assay (Figure S1). For comparison, 5% DMSO was used as the control for high cellular toxicity.

To visualize the endocytic pathway, C6 cells were incubated with DiI-labeled LDL (20 µg/mL of cholesterol), as previously described [10]. Treatment with 2.5 µM U18666A (control) for 16 h resulted in intense DiI-LDL staining in bright perinuclear granules, which were positive for the endolysosome marker, CD63, indicating that U18666A affected the release of LDL-cholesterol from the endosome/lysosome compartment in this cell line [10]. Then, to study the effects of rottlerin, the cells treated with U18666A were exposed to rottlerin for the last 4 h of incubation.

**Figure 1:**
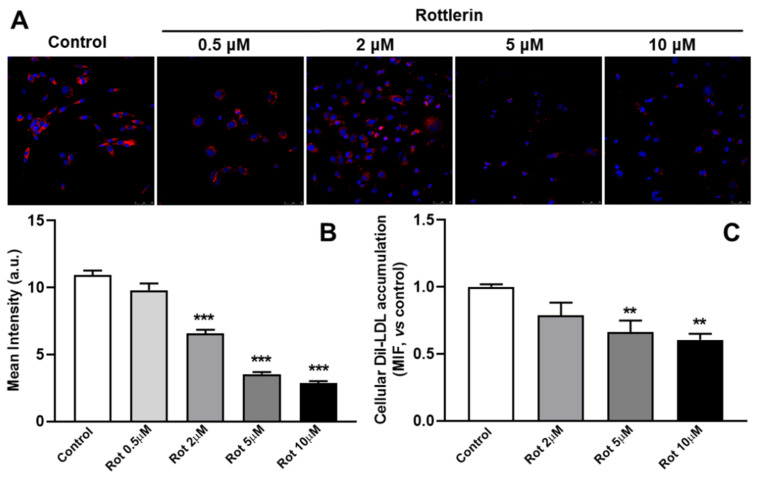
Effects of rottlerin on the intracellular distribution of LDL in cells treated with U18666A. (**A**) C6 cells were exposed to DiI-LDL (20 μg/mL of cholesterol) and 2.5 μM U18666A for a total of 16 h without (control) or with rottlerin (0.5 μM, 2 μM, 5 μM, and 10 μM) for the last 4 h. The cells were then washed, fixed and analyzed by confocal microscopy. Images show DiI-LDL in red and nuclei labeled with Hoechst-33342 in blue. Representative results from three independent experiments are shown. Scale bars = 25 μm. (**B**) The graph shows the results of the quantification of DiI-LDL (mean intensity, a.u.) of cells of each photomicrograph. Results are the mean ± SEM of three independent experiments, with 8−10 photographs per experiment. (**C**) The graph represents the intracellular DiI-LDL fluorescence in C6 cells exposed to DiI-LDL (20 μg/mL of cholesterol) and U18666A (2.5 μM) for a total of 16 h with or without (Control) treatment with rottlerin (2 μM, 5 μM, and 10 μM) for the last 4 h, by flow cytometry. The control is normalized to 1. Results are the mean ± SEM of three independent experiments. Rot, rottlerin. Statistical comparisons shown are rottlerin versus control ( ** *p* < 0.01, *** *p* < 0.001).

Rottlerin induced a dose-dependent decrease in the endo/lysosomal accumulation of DiI as measured by immunofluorescence (Figure 1A,B). Similar results were found when intracellular DiI-LDL levels were measured by flow cytometry (Figure 1C). The results suggested that rottlerin counteracted the accumulation of DiI-LDL in endo/lysosomes exerted by U18666A, and inhibitor of Niemann–Pick type C1 (NPC1) [32].

### 3.2. Rottlerin Stimulates Exosome/Microvesicle Secretion

**Figure 2:**
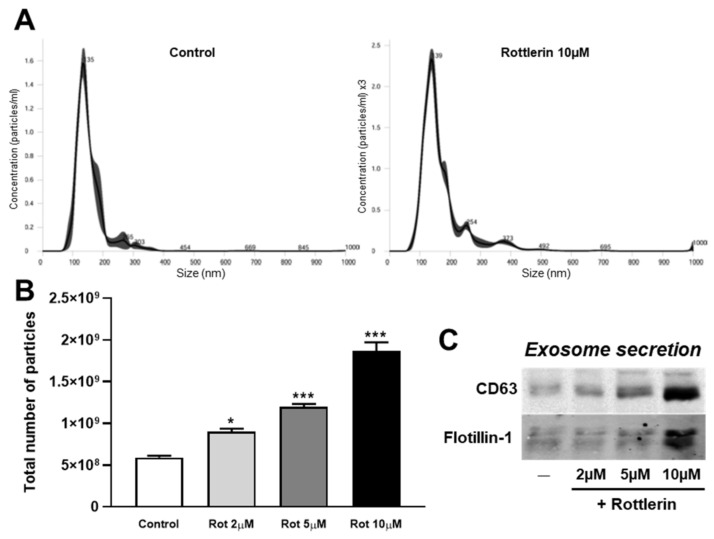
Rottlerin enhances the release of exosomes/microvesicles from C6 cells. The experiments started with the same number of cells. Cells were exposed to LDL (60 µg/mL of cholesterol) in the presence of 2.5 µM U18666A for 12 h. The medium was then removed, cells were washed twice, and serum-free medium was added, supplemented with U18666A, and the incubation was continued for 4 h in the presence of increasing doses of rottlerin (2 μM, 5 μM, or 10 μM, Rot). At the end of the incubation, the medium was removed, and the exosomes/microvesicles were isolated and analyzed. (**A**) Exosomes/microvesicles from C6 cells in control conditions or treated with rottlerin were isolated and subjected to nanoparticle tracking analysis. The graphs represent the size (nm) of the exosomes/microvesicles. (**B**) The graph represents the total number of particles per experiment by nanoparticle tracking analysis. Means ± SEM of three measures. (**C**) Flotillin-1 and CD63 Western blots of exosomes/microvesicles isolated from C6 cells. Results shown are representative of three independent experiments. Statistical comparisons of treatments versus the control (* *p* < 0.05, *** *p* < 0.001).

The mentioned effects of rottlerin were reminiscent of those exerted by curcumin, another polyphenol, which decreases cellular accumulation of DiI-LDL by stimulating exosome/microvesicle secretion [10]. To ascertain this possibility, C6 cells were exposed to LDL (60 µg/mL of cholesterol) in the presence of 2.5 µM U18666A for 16 h. The medium was then removed, the cells washed twice, and serum-free medium supplemented with U18666A was added, and the incubation was continued for 4 h in the absence (Control) or the presence of increasing doses of rottlerin (2, 5, and 10 µM). At the end of incubation, the medium was removed and the exosomes/microvesicles were isolated, and the size and specific protein markers were analyzed by NanoSight and Western blotting, respectively (Figure 2). The size of secreted exosomes/microvesicles from C6 cells treated with U18666A in the absence of rottlerin (control) was 141.3 ± 2.6 nm (mean ± SEM), similar to those in cells treated with 10 µM rottlerin (Figure 2A). Similar results were found for 2 µM and 5 µM rottlerin (data not shown).

We also examined the number of secreted exosomes/microvesicles using NanoSight. The results are shown in Figure 2B, which corresponded to the total secretion of exosomes/microvesicles per condition, all starting with the same number of cells in the well. The number of exosomes/microvesicles secreted from 10 µM rottlerin-treated cells was three times higher than that from cells incubated in control conditions. Moreover, rottlerin enhanced the secretion of the exosome/microvesicle protein markers, CD63 and flotillin-1, in the isolated fraction (Figure 2C).

### 3.3. Rottlerin Alters Sphingolipid Cell Content and Composition

We previously reported that curcumin, the main active polyphenol extracted from the rhizome of *Curcuma longa*, stimulates exosome/microvesicle release by increasing the intracellular concentration of ceramide [10]. We determined the levels of sphingolipids (ceramide, hexosylceramide, and sphingomyelin) in glioma C6 cells treated with or without increasing doses of rottlerin for 4 h (Table 1). The levels of ceramide significantly increased after 4 h of treatment with rottlerin, while hexosylceramide and sphingomyelin levels decreased. In more detail, in cells treated with rottlerin Cer 34:1 and Cer 36:1 species increased, while HexCer 34:1 and SM 34:1 decreased compared to control cells (Figure S2). Thus, the increase of ceramide, in detriment of the other sphingolipids, may be responsible for the stimulation of exosome/microvesicle release by rottlerin.

### 3.4. Rottlerin Alters Galactosylceramidase and Glucosylceramidase Activities

**Figure 3:**
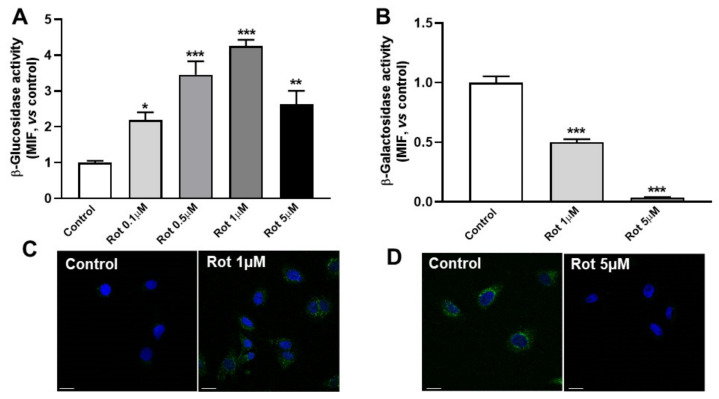
Rottlerin alters β-galactosidase and β-glucosidase activities. C6 cells were incubated in LPDS medium for 2 h, then treated with or without (Control) rottlerin (Rot), then the reaction substrates for β-galactosidase (C12FDG) or β-glucosidase (FDGlu) were added for the last 30 min and analyzed by flow cytometry or immunocytochemistry. (**A**) The β-glucosidase activity analyzed by flow cytometry. (**B**) The β-galactosidase activity analyzed by flow cytometry. The control is normalized to 1. Results are the mean ± SEM of three independent experiments. MIF, median intensity of fluorescence. Statistical comparisons shown are treatment versus control (* *p* < 0.05, ** *p* < 0.01, *** *p* < 0.001). (**C**) Immunocytochemistry of β-glucosidase after treatment with or without rottlerin. (**D**) Immunocytochemistry of β-galactosidase activity after treatment with or without rottlerin. Images show the reaction product in green, and the nuclei labeled with Hoechst-33342 in blue. Representative results from three independent experiments are shown. Scale bars = 25 μm.

We analyzed the activities of enzymes that degrade hexolsylceramides after rottlerin treatment, involving galactosylceramidase (β-galactosidase) and glucosylceramidase (β-glucosidase). As shown in Figure 3A,B, β-galactosidase activity significantly decreased after treatment with rottlerin in C6 cells, while β-glucosidase increased up to four times that in control conditions. Similar results were found when these enzymatic activities were analyzed by immunocytochemistry (Figure 3C,D). Together, the results showed that treatment with rottlerin decreased hexosylceramide content by increasing β-glucosidase activity.

### 3.5. Inhibition of β-Glucosidase Prevents the Effects of Rottlerin on Exosome/Microvesicle Release and Cellular Sphingolipid Levels

**Figure 4:**
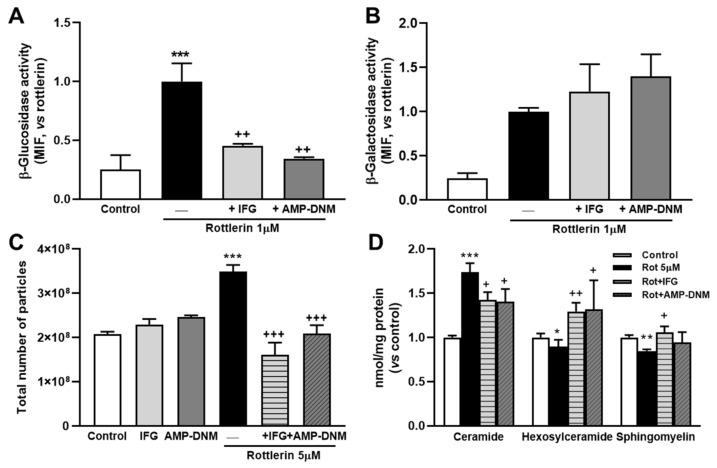
Effect of β-glucosidase inhibitors on rottlerin effects in a model of impaired intracellular lipid trafficking. (**A**) The graph represents β-glucosidase activity. C6 cells were incubated in LPDS medium in the presence of AMP-deoxynojirimycin (AMP-DNM) or isofagomine D-tartrate (IFG) for 2 h, then treated with or without (Control) rottlerin (Rot), and the reaction substrates for β-galactosidase (C12FDG) or β-glucosidase (FDGlu) were added for the last 30 min and finally analyzed by flow cytometry. (**A**) β-glucosidase activity. (**B**) β-galactosidase activity. Results are the mean ± SEM of three independent experiments. The results with rottlerin are normalized to 1. MIF, median intensity of fluorescence. (**C**) Total number of particles per experiment analyzed by nanoparticle tracking. The experiments started with the same number of cells in all the conditions. Means ± SEM of three measures. (**D**) Sphingolipid cell contents. Cells were incubated without (Control) or with 5 μM rottlerin (Rot) in combination with or without AMP-DNM or IFG for 4 h. Ceramides, hexosylceramides, and sphingomyelin were measured by mass spectrometry as described in Methods. Results are the mean ± SEM of three independent experiments. The control is normalized to 1. Statistical comparisons shown are rottlerin versus control (* *p* < 0.05, ** *p* < 0.01, *** *p* < 0.001) or β-glucosidase inhibitor plus rottlerin versus rottlerin alone (^+^ *p* < 0.05, ^++^ *p* < 0.01, ^+++^ *p* < 0.001).

To inhibit β-glucosidase, we used IFG [33] and AMP-DNM [34,35]. The cells were cultured in LPDS medium in the presence of AMP-DNM or IFG for 2 h, then treated with or without rottlerin with the substrate for β-galactosidase or β-glucosidase enzymes added for the last 30 min. Both inhibitors, IFG and AMP-DNM, significantly prevented the increase in activity of β-glucosidase by rottlerin (Figure 4A). However, no significant change in β-galactosidase activity by these inhibitors was observed (Figure 4B).

We also examined the effects of β-glucosidase inhibitors on the number of secreted exosomes/microvesicles by using NanoSight. The results are shown in Figure 4C and correspond to the total secretion of exosomes/microvesicles per condition, which all started with the same number of cells in the wells. The β-glucosidase inhibitors had no detectable effect on the release of exosome/microvesicle particles in cells not treated with rottlerin. However, for cells treated with rottlerin, the addition of IFG or AMP-DNM totally abolished the effect of the phytochemical (Figure 4C).

Supplementation with IFG or AMP-DNM significantly reduced ceramide levels in cells treated with rottlerin (Figure 4D). In contrast, hexosylceramide levels increased after treatment with β-glucosidase inhibitors (Figure 4D). The reduction of sphingomyelin levels by rottlerin was also prevented by IFG, but not as strongly by AMP-DNM (Figure 4D). Taken together, these results suggested that the increase in ceramide content induced by rottlerin was due, at least in part, to the degradation of hexosylceramides, especially glucosylceramides, through the activation of β-glucosidase.

### 3.6. Activation of Enzyme β-Glucosidase Stimulates Exosome/Microvesicle Release through the Increase in Ceramide

**Figure 5:**
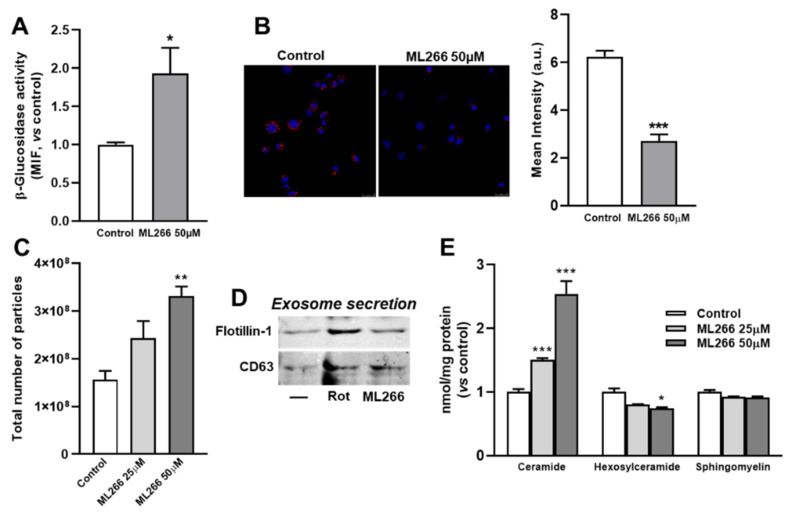
Effects of β-glucosidase activator on glioma C6 cells (**A**) β-glucosidase activity as analyzed by flow cytometry. Cells were incubated in LDL-supplemented medium with or without ML266 for 2 h, then the reaction substrate was added for 30 min and analyzed by flow cytometry. Results are the mean ± SEM of three independent experiments. The control is normalized to 1. MIF, median intensity of fluorescence. (**B**) C6 cells were exposed to DiI-LDL (20 μg/mL of cholesterol) and 2.5 μM U18666A for a total of 16 h and without (Control) or with ML266 (50 μM) for the last 4 h. The cells were fixed and analyzed by confocal microscopy. The images show DiI-LDL in red and nuclei labeled with Hoechst-33342 in blue. Representative results from three independent experiments are shown. Scale bars = 25 μm. The graph shows quantitative results of DiI-LDL labeling (mean intensity, a.u.) in cells of each photomicrograph. Results are the mean ± SEM of three independent experiments, with 8−10 photographs per experiment. (**C**) Total particle secretion analyzed by nanoparticle tracking. The experiments started with the same number of cells. Cells were exposed to LDL (60 µg/mL of cholesterol) in the presence of 2.5 µM U18666A for 12 h. The medium was then removed, the cells washed twice, and serum-free medium was added, supplemented with U18666A, then incubation was continued for 4 h in the presence of 50 μM ML266 or 5 μM rottlerin (Rot). At the end of the incubation, the medium was removed, and the exosomes/microvesicles were isolated and analyzed. The mean ± SEM of three measures are shown. (**D**) Western blot of flotillin-1 and CD63 in secreted particles. Results are representative of three independent experiments. (**E**) Sphingolipids cell contents. Cells were incubated without (Control) or with 50 μM ML266 for 4 h. Ceramides, hexosylceramides, and sphingomyelin were measured by mass spectrometry as described in Methods. Results are the mean ± SEM of three independent experiments. The control is normalized to 1. Statistical comparisons shown are treatment versus control (* *p* < 0.05, ** *p* < 0.01, *** *p* < 0.001).

Prompted by the results shown above, we determined whether selective activation of β-glucosidase caused similar effects as rottlerin on lysosomal lipid accumulation and exosome/microvesicle release. ML266 is a chemical chaperone that stabilizes β-glucosidase and facilitates its translocation to lysosomes [36,37]. Figure 5A shows that treatment with ML266 significantly increased β-glucosidase activity. On the other side, DiI levels decreased when cells incubated with U18666A were further treated with ML266 for 4 h (Figure 5B). Moreover, ML266 enhanced the secretion of exosomes/microvesicles as determined using NanoSight (Figure 5C) as well as the protein markers, flotillin-1 and CD63 (Figure 5D). Finally, treatment with ML266 caused a dose-dependent increase in cellular levels of ceramide, while decreasing those of hexosylceramide without affecting those of sphingomyelin (Figure 5E).

Thus, in a model of intracellular lipid trafficking, chemical activators of β-glucosidase increased the amount of ceramide through the degradation of hexosylceramide and stimulated exosome/microvesicle release, facilitating the export of lipids out of the cell, thereby ameliorating the endo/lysosomal lipid accumulation found in lysosomal storage disorders.

### 3.7. Effects of Rottlerin on the Gene Expression of Enzymes Involved in Glucosylceramide Metabolism

Given that treatment with rottlerin increased glucosylceramidase activity and ceramide content in cells, we measured the mRNA levels of lysosomal β-glucosylceramidase (Gba), non-lysosomal β-glucosylceramidase (Gba2), and glucosylceramide synthase (Ugcg) in C6 cells treated with or without rottlerin. No significant change in the expression of the Gba gene relative to control cells was observed, while Gba2 and Ugcg gene expressions decreased after 4 h of treatment with both doses of rottlerin (Figure S3).

### 3.8. The Role of the AMPK Pathway on Rottlerin Effects on Exosome Secretion

**Figure 6:**
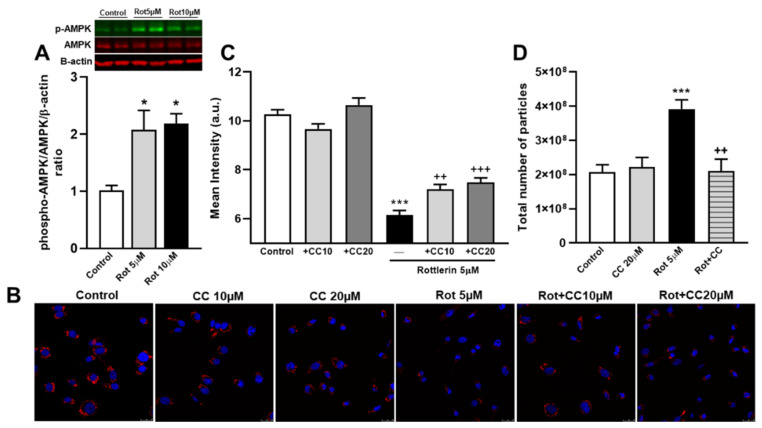
Rottlerin stimulates exosome/microvesicle release via activation of the AMPK (AMP-activated protein kinase) pathway. (**A**) Western blotting and densitometric analyses of whole-cell lysates using anti-phospho-AMPK (T172), AMPK (total), and β-actin antibodies in C6 cells exposed to LDL (20 μg/mL of cholesterol) and 2.5 μM U18666A for a total of 16 h without (Control) or with rottlerin (5 μM and 10 μM, Rot) for the last 4 h. Results are the mean ± SEM of three independent experiments. Representative immunoblots are shown. (**B**) C6 cells were exposed to DiI-LDL (20 μg/mL of cholesterol) and U18666A for a total of 16 h without (Control) or with rottlerin in combination with or without compound C (10 μM or 20 μM, CC) for the last 4 h. The cells were then washed, fixed, and analyzed by confocal microscopy. Images show DiI-LDL in red and nuclei labeled with Hoechst-33342 in blue. Representative results from three independent experiments are shown. Scale bars = 25 μm. (**C**) The graph shows quantitative results of DiI-LDL labelling (mean intensity, a.u.) in cells of each photomicrograph. Results are the mean ± SEM of three independent experiments, with 8−10 photographs per experiment. (**D**) Total particle secretion analyzed by nanoparticle tracking. The experiments started with the same number of cells. Cells were exposed to LDL (60 µg/mL of cholesterol) in the presence of 2.5 µM U18666A for 12 h. Then, the medium was removed, cells were washed twice, and serum-free medium was added, supplemented with U18666A, and incubation was continued for 4 h with or without 5 μM rottlerin (Rot) in combination with or without 20 μM compound C (CC). At the end of the incubation, the medium was removed, and the exosomes/microvesicles were isolated and analyzed. The graphs represent the total number of particles per experiment analyzed by nanoparticle tracking. Means ± SEM of three measures. Statistical comparisons shown are rottlerin versus control (* *p* < 0.05, *** *p* < 0.001) or compound C plus rottlerin versus rottlerin alone (^++^ p < 0.01 and ^+++^ p < 0.001).

It has been previously reported that rottlerin activated the AMPK pathway in cells [21,22]. In our cell system, we found that treatment with rottlerin (5 and 10 µM) for 4 h also activated AMPK, as indicated by the increase of phosphorylated AMPK (T172) (Figure 6A). Thus, we determined the possible involvement of AMPK in exosome/microvesicle secretion induced by rottlerin. To accomplish this objective, we tested the effect of compound C, a selective and ATP-competitive AMPK inhibitor, in C6 cells. In cells not treated with rottlerin, compound C did not affect the intracellular content of DiI (Figure 6B,C) or the secretion of exosome/microvesicles (Figure 6D). In contrast, in cells exposed to rottlerin, compound C partially counteracted the elimination of DiI from lysosomes (Figure 6B,C) and abrogated the secretion of particles to the medium (Figure 6 D). Taken together, these findings indicated that AMPK activation was involved in the rottlerin-induced elimination of lipids, which accumulated in lysosomes, via exosome/microvesicle secretion.

## 4. Discussion

The mechanism responsible for the stimulation of exosome/microvesicle release by rottlerin was studied in a cell model of late endosomal/lysosomal lipid accumulation. We showed that rottlerin increased ceramide levels through degradation of hexosylceramides via the activation of β-glucosidase (lysosomal glucosylceramidase β), thus stimulating exosome/microvesicle release. This effect of rottlerin was mediated by AMPK activation.

Glioma C6 cells have the ability to intensely synthesize sphingolipids, and therefore represent a suitable cell model to search for ways to ameliorate perturbations caused by intracellular lipid trafficking disruption relevant in the central nervous system. To overload the endolysosome compartment with lipids, C6 cells were incubated in the presence of LDL and treated with U18666A to inhibit NPC1 [31]. We found that treatment with rottlerin decreased the size of endolysosomes previously accumulated in these cells, which may be attributed to the observed stimulation of exosome/microvesicle release to the medium. Strauss et al. first described the increase of exosomal cholesterol secretion in NPC1 deficient fibroblasts, which ameliorated the accumulation of cholesterol in lysosomes [38]. In our previous studies, we reported an increase of exosomal lipid secretion after treatment with curcumin in different models of endolysosome lipid trafficking blockade [10,25,39]. We propose that the egress of lipids out of the cell induced by rottlerin ameliorated lipid retention within the endolysosomal compartment to maintain cellular lipid homeostasis.

To elucidate the mechanism responsible for the effects of rottlerin, we characterized several pathways. Based on previous studies by Kosaka et al. showing that rottlerin increased ceramide concentrations after long treatments [17], and our results on the action of other drugs and compounds on intracellular lipid trafficking [10,40], we first focused on sphingolipid metabolism. We showed that rottlerin treatment increased ceramide content in C6 cells, together with the degradation of hexosylceramides. Consistent with this observation, rottlerin increased β-glucosidase activity in a dose-dependent manner, while inhibiting β-galactosidase (galactosylceramidase) activity. Treatment with β-glucosidase inhibitors, IFG or AMP-DNM, reversed the effects of rottlerin, because they counteracted the ceramide increase and hexosylceramide reduction induced by the polyphenol. Importantly, both IFG and AMP-DNM prevented exosome/microvesicle release induced by rottlerin. Moreover, activation of β-glucosidase by ML266 recapitulated the effects of rottlerin on the sphingolipid profile and exosome/microvesicle secretion. Taken together, these results strongly suggested that the effects of rottlerin were mediated by β-glucosidase activation.

The mechanism involved in the rottlerin-activation of β-glucosidase is not known, given that gene expression of Gba (lysosomal glucosylceramidase β or β-glucosidase) was not affected. In contrast, rottlerin inhibited the gene expression of Gba2 (non-lysosomal glucosylceramidase β 2) and Ugcg (UDP-glucose ceramide glucosyltransferase or glucosylceramide synthase) (Figure S4). This is consistent with recent results by Honda et al., who reported that rottlerin treatment inhibited the formation of glucosylceramide in HeLa-v-Src cells [41]. Therefore, rottlerin could reduce glucosylceramide levels by inhibiting its formation via down-regulation of Ugcg expression and increasing their degradation via β-glucosidase activation.

GBA2 is a non-lysosomal glucosylceramidase β that hydrolyzes glucosylceramide and also bile acid-3-O-β-glucosides [42]. GBA2 is a non-integral membrane protein that localizes at the cytosolic surface of the endoplasmic reticulum and Golgi, where it is in close association with membrane phospholipids [43], and where it is thought to control the amount of newly synthesized cytosolic glucosylceramide [42]. However, the role, if any, of this enzyme on lysosomal lipid metabolism has not been established yet.

In animal cells, the main site for degradation of glucosylceramide and galactosylceramide involves lysosomes. Hexosylceramides, after endocytosis of plasma membrane fragments, are transported to the lysosome by the endocytic vesicular pathway. Glycosphingolipids are then degraded on the surface of intralysosomal vesicles [44]. In addition to the enzymes, essential components of the intra-lysosomal degradation of glycosphingolipids are the sphingolipid activator proteins, saposins A, B, C and D and GM2-activator protein [44]. It has been proposed that saponins activate glycosphingolipid degradation by either (1) facilitating the interaction between membrane-localized glycosphingolipids and these water-soluble exohydrolases, or (2) binding directly to enzymes, thus generating a more active enzymatic complex [44]. Notably, deficiency of saposin C may lead to accumulation of glucosylceramide within cells, resulting in a glucosylceramide storage disease and a neurological form of Gaucher disease, which shows the crucial role of this cofactor in the metabolism of this hexosylceramide [45]. However, we cannot exclude the possibility that the polyphenol rottlerin exerts its effects through sphingolipid activator proteins.

Several studies have reported the role of ceramide in exosome/microvesicle release [7,8,9,10,11,46]. The specific inhibition of neutral SMase has been shown to reduce the secretion of exosomes [13]. Mechanistically, ceramides can induce lateral phase separation and spontaneous curvature of membranes, thereby promoting endosome budding [12,13]. Based on these results, we propose that rottlerin, by increasing ceramide content, stimulates the release of exosome/microvesicles particles out of the cell, thus reducing the intracellular lipid deposition. Whether other mechanisms essential in the intra-lysosomal degradation of lipids and the exosome/microvesicles formation and release are also affected by rottlerin cannot be eliminated.

Treatment with rottlerin induced AMPK phosphorylation in glioma C6 cells. Similar results were reported in prostate cancer stem and neuroblastoma cells [21,22]. AMPK is a master controller of metabolic homeostasis and plays a central role in glucose and lipid metabolism [18]. It has been shown that activation of AMPK decreased intracellular glucosylceramide levels and synthase activity by reducing intracellular levels of UDP-glucose in mouse fibroblasts [47]. However, AMPK phosphorylation sites have not been identified in the amino acid sequences of GBA and GBA2 [47]. Recently, Lee et al. found a link of AMPK with sphingolipid metabolism in platelets, because the increase in sphingomyelin level in H_2_O_2_-treated platelets was prevented by AMPK inhibitor compound C [48]. The AMPK pathway has been also linked to exosome/microvesicle secretion [19,20]. In cultured adipocytes and white adipose tissue, the absence of AMPKa1 increased exosome release, and AMPK activation by metformin reduced adipocyte-mediated exosome release [19]. However, inhibition of AMPK has been recently shown to reduce exosome secretion induced by thioredoxin in retinal photoreceptor cells [20]. In the present study, inhibition of AMPK by compound C reversed rottlerin-promoted exosome/microvesicle secretion and reduction of lipid accumulation in endolysosomes.

**Figure 7:**
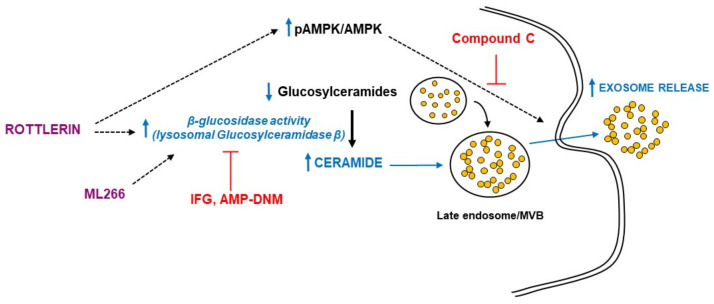
Summary of the rottlerin effects in vitro model of intracellular lipid accumulation. Rottlerin stimulated exosome/microvesicle release via activation of β-glucosidase that degraded glucosylceramide, increasing ceramide levels, which facilitated the export of lipids out of the cell and thus ameliorated the endo/lysosomal lipid accumulation in a model of intracellular lipid trafficking impairment in glioma cells. AMPK (AMP-activated protein kinase) activation mediated the effects of rottlerin. ML266 is a chemical chaperone that stabilizes β-glucosidase and facilitates its translocation to lysosomes. Compound C is a selective and ATP-competitive AMPK inhibitor. IFG (isofagomine D-tartrate) and AMP-DNM (AMP-deoxynojirimycin) inhibit the β-glucosidase. MVB, multivesicular bodies.

In conclusion, the present study showed that in a model of intracellular lipid trafficking impairment in glioma cells, rottlerin stimulated exosome/microvesicle release via activation of β-glucosidase that degraded glucosylceramide, increasing ceramide levels, which facilitated the export of lipids out of the cell and thus ameliorated the endo/lysosomal lipid accumulation found in lysosomal storage disorders (Figure 7). AMPK activation mediated the effects of rottlerin. This action may represent an additional health benefit of rottlerin consumption.

## Figures and Tables

**Table 1:** Effect of rottlerin on sphingolipid contents. Cells were incubated without (Control) or with increased doses of rottlerin (2 μM, 5 μM, and 10 μM) for 4 h. Ceramides, hexosylceramides, and sphingomyelin were measured by mass spectrometry. Results are the mean ± SEM from three independent experiments, expressed in nmol/mg protein. Statistical comparisons of treatments versus control (* *p* < 0.05, ** *p* < 0.01, *** *p* < 0.001).

Sphingolipid	Control		Rottlerin	
		2 μM	5 μM	10 μM
Ceramide	0.71 ± 0.04	0.97 ± 0.03 **	0.91 ± 0.02 **	0.91 ± 0.04 **
Hexosylceramide	2.18 ± 0.05	1.85 ± 0.06 **	1.82 ± 0.02 **	1.70 ± 0.01 ***
Sphingomyelin	20.13 ± 1.28	18.77 ± 0.16	17.49 ± 0.35 **	18.09 ± 1.07 *

## Data Availability

Data are contained within the article or Supplementary Materials. The datasets generated and analyzed during the current study are available from the corresponding author upon reasonable request.

## Supplementary Materials

The following supporting information can be downloaded at: https://www.mdpi.com/article/10.3390/biomedicines10061316/s1, Figure S1: Effects of the drugs used on cell viability C6 cells; Figure S2. Effect of rottlerin on sphingolipids species content; Figure S3. Effects of rottlerin gene expression of enzymes involved in glucosylceramide metabolism; Figure S4. An overview of the sphingolipid metabolism pathway; Table S1. Sequence of PCR primers used in quantitative real-time PCR.
