# Supplementary material for: Rottlerin Stimulates Exosome/Microvesicle Release Via the Increase of Ceramide Levels Mediated by Ampk in an In Vitro Model of Intracellular Lipid Accumulation
Source: Biomedicines. 2022 Jun 3;10(6):1316. doi: 10.3390/biomedicines10061316 (PMC9219951; doi:10.3390/biomedicines10061316)
Supplement: Supplementary file 1 [file biomedicines-10-01316-s001.zip › biomedicines-1713605-supplementary.pdf]

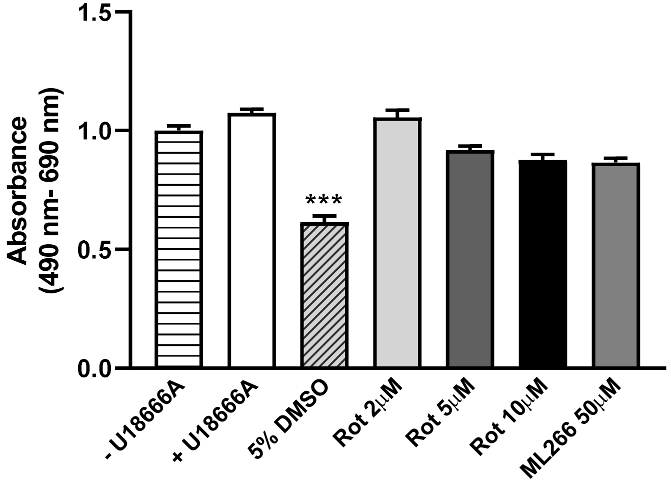

Figure S1. Effects of the drugs used on cell viability inC6 cells. Cells were exposed or not to 2.5 μM U18666A, rottlerin (2, 5 or 10 μM), or ML266 (a β-glucosidase activator) for 4 h, respectively. Then, the mitochondrial activity (XTT assay) was measured after the treatment. Values are the mean ± S.E.M of three independent experiments with three replicates each. Statistical comparisons versus control are shown (***P<0.001).

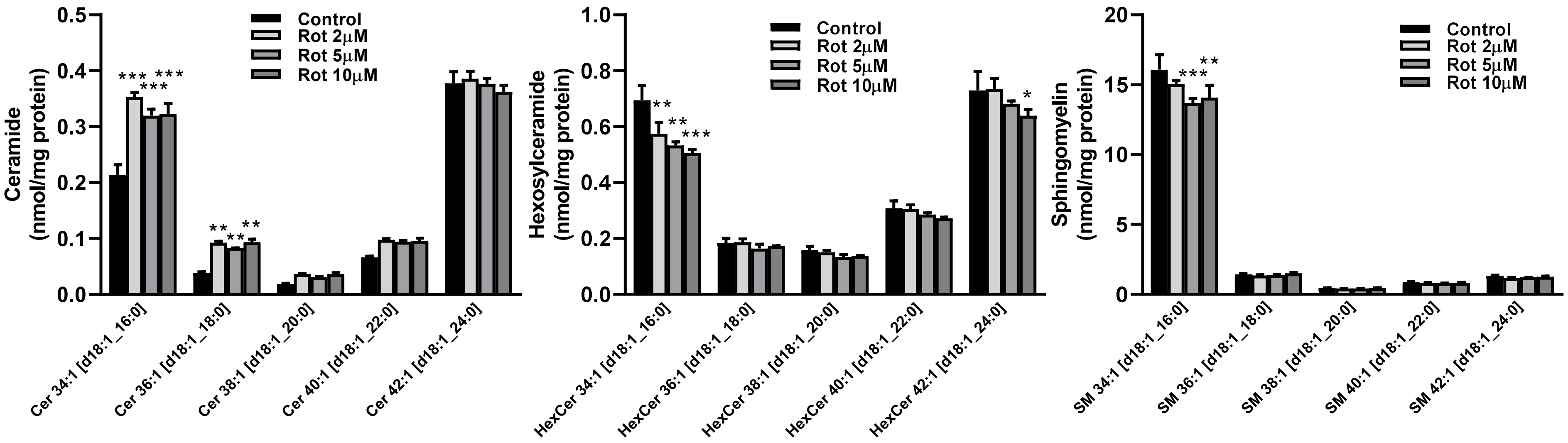

Figure S2. Effect of rottlerin on sphingolipids species content. Cells were incubated without (Control) or with increased doses of rottlerin (2, 5 and 10 μM, Rot) during 4 h. Ceramides, hexosylceramides and sphingomyelin were measured by MS as described in Methods. Results are mean ± SEM from 3 independent experiments. Statistical comparisons of treatments versus control are shown (** $P < 0.01$ and *** $P < 0.001$).

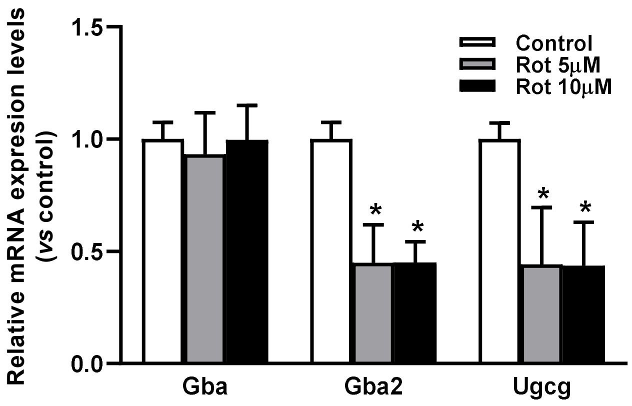

Figure S3. Effects of rottlerin on gene expression of enzymes involved in glucosylceramide metabolism. C6 cells were treated or not with rottlerin for 2 h or 4 h. At the indicated times, cells were collected, total mRNA was extracted, and individual mRNA species were quantified by qRT-PCR. Expression levels were normalized to the media of Gapdh and Rplp0 mRNA. Expression is presented as means ± SEM of three independent experiments performed in triplicate. The control is normalized to 1.

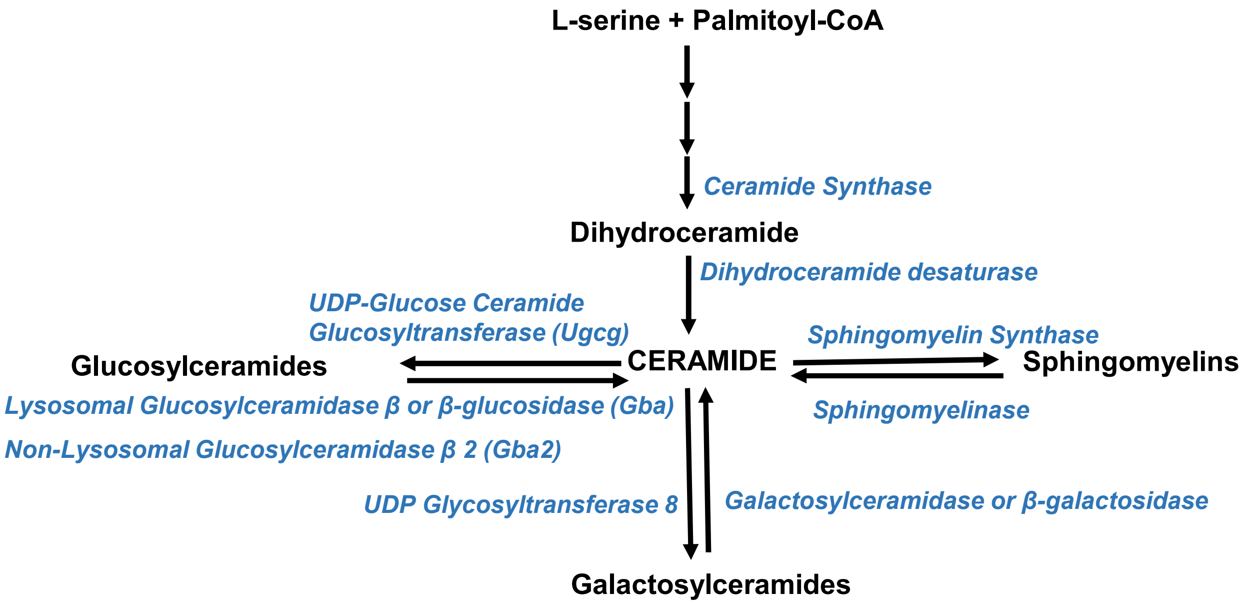

Figure S4. An overview of the sphingolipid metabolism pathway.

Table S1. Sequence of PCR primers used in quantitative real-time PCR.

Gene	Gen bank ID	Forward Primer	Reverse Primer
Gba	NM_001127639	5'-GAGCAGAGTGTTCGGTTAGG-3'	5'-GATTCAGGGCAAGGTTCCAG-3'
Gba2	NM_001013091	5'-TCGACATGTTCAATTCTGTACCC-3'	5'-AAGCTGCCAACGACAGAACT-3'
Ugcg	NM_031795	5'-TCCGATGGGATATCATGGTT-3'	5'-TGAACCAAGCCACAGCATAA-3'
Rplp0	NM_022402	5'-GGCGACCTGGAAGTCCAACT-3'	5'-CCATCAGCACCACAGCCTTC-3'
Gapdh	NM_017008	5'-AGGTCGGTGTGAACGGATTTG-3'	5'-TGTAGACCATGTAGTTGAGGTCA-3'
